# Book Reviews

**Published:** 1904-11

**Authors:** 


### BOOK REVIEWS.

Dental Metallurgy: A Manual for the use of Dental Students and Practitioners. By Charles J. Essig, M. D., D. D. S., and Augustus Koenig, B. S., M. D. Fifth Edition, revised and enlarged, 318 pages, with 76 engravings. Philadelphia and New York: Lea Bros & Co. 1904. Price $2.00 net.

Essig's Metallurgy has been before the profession for many years and has come to be accepted as the standard work upon the subject.

The late Dr. Essig recognized the need of a work of this kind and in the first edition compiled all the available data on the subject, together with the results of his own work, and Dr. Koenig in the present edition has added valuable material which brings it down to a level with the present state of advancement in this line. Secure a copy and study it.

DUNHAM'S NORMAL HISTOLOGY: A TEXT-BOOK OF NORMAL HISTOLOGY FOR THE USE OF STUDENTS AND PRACTITIONERS OF MEDICINE. By Edward K. Dunham, Ph. B., M. D. Professor of General Pathology, Bacteriology, and Hygiene in the University and Bellevue Hospital Medical College, New York. New (3rd) edition, revised and enlarged. In one octavo volume of 334 pages, with 260 illustrations. Philadelphia and New York: Lea Bros & Co. 1904. Price $2.75 net.

The author states in his preface that the general plan and scope of this outline of histology are the result of experience in teaching the subjects to students of medicine under conditions which require economy of time. The book is divided into nineteen chapters and in their contents the student will find accurate and descriptive information on the microscopical anatomy of all the tissues of the body.

It is probable that the work has been boiled down a trifle more than is conducive to a ready understanding and this is clear when it is stated that the whole subject of the structure of the teeth is covered in less than two pages.

On the whole the book should prove of value to medical students and to dental students as well in connection with the subject of general histology and the practitioner of medicine can read it with profit. The makeup of the work is good —in harmony with the well known standards of the publishers.

THE DOCTOR'S LEISURE HOUR—FACTS AND FANCIES OF INTEREST TO THE DOCTOR AND HIS PATIENT—ARRANGED By Porter, Davies, M. D., Volume 1 of The Doctor's Recreation Series, Charles Wells Moulton, General Editor. Pages 352, Illustrated, Silk Cloth, $2.50 The Saalfield Publishing Co, Akron, O.

The Doctor's Leisure Hour, contains a fund of interesting matter relating to the lighter side of the practice of medicine. It consists of stories, sketches, and poems covering the entire life of the doctor from student days until he becomes the "leading doctor of the town."

Anecdotes of famous doctors, poems on Madame La Docteur, sketches and jokes of the quack, etc., abound. The volume contains a great wealth of wit and humor but also has some serious matter. The selections are from the note-books of an active practitioner and are made under the supervision of the general editor. Among the contributors are Dr. S. Weir Mitchell, John Kendrick Bangs, Opie Read, Jerome K. Jerome, Alice Morse Earle, Rev. John Watson, Ruth McEnery Stuart, Mary E. Wilkins, MacKenzie Bell, Edwin L. Sabin.

The book is well printed on a good quality of paper and well bound in silk cloth with gilt top. The Doctor's Recreation Series will consist of twelve volumes, and will constitute an actual encyclopedia of purely literary medical literature.

